# Supplementary material for: Distinguishing Invasive from Chronic Pulmonary Infections: Host Pentraxin 3 and Fungal Siderophores in Bronchoalveolar Lavage Fluids
Source: J Fungi (Basel). 2022 Nov 12;8(11):1194. doi: 10.3390/jof8111194 (PMC9694386; doi:10.3390/jof8111194)
Supplement: Supplementary file 1 [file jof-08-01194-s001.zip › jof-1944834-supplementary.pdf]

Article

Distinguishing invasive from chronic pulmonary infections: host pentraxin 3 and fungal siderophores in bronchoalveolar lavage fluids

Radim Dobiáš [1,2]*, Pavla Jaworská [1], Valeria Skopelidou [2], Jan Strakoš [2], Denisa Višňovská [2,3], Marcela Káňová [4,5,6], Anton Škríba [7], Pavlína Lysková [8], Tomáš Bartek [9], Ivana Janíčková [9], Radovan Kozel [10], Lucie Cwiková [10], Zbyněk Vrba [11], Milan Navrátil [12], Jan Martinek [13,14], Pavla Coufalová [15], Eva Krejčí [1,2], Vít Ulmann[1,16], Milan Raška [17], David A. Stevens [18,19] and Vladimír Havlíček [7,20]

[1] Department of Bacteriology and Mycology, National Reference Laboratory for Mycological Diagnostics, Public Health Institute in Ostrava, 702 00 Ostrava, Czechia
[2] Institute of Laboratory Medicine, Faculty of Medicine, University of Ostrava, 703 00 Ostrava, Czechia
[3] Department of Biology and Ecology, Faculty of Science, University of Ostrava, 710 00 Ostrava, Czechia
[4] Department of Anesthesiology and Intensive Care Medicine, University Hospital Ostrava, 708 00 Ostrava
[5] Institute of Physiology and Pathophysiology, Faculty of Medicine, University of Ostrava, 710 00 Ostrava, Czechia
[6] Department of Intensive Medicine, Emergency Medicine and Forensic Studies, University of Ostrava, 710 00 Ostrava, Czechia
[7] Institute of Microbiology of the Czech Academy of Sciences, 142 20 Prague, Czechia
[8] Department of Medical Microbiology Prague and Kladno, Public Health Institute in Ústí nad Labem, 186 00 Prague, Czech Republic
[9] Department of Lung Disease and Tuberculosis, University Hospital Ostrava, 708 00 Ostrava, Czechia
[10] Department of Pneumology and Phthisiology, Ostrava City Hospital, 728 80 Ostrava, Czech Republic
[11] Lung Department, Krnov Combined Medical Facility, 794 01 Krnov, Czech Republic
[12] Department of Hemato-oncology, University Hospital of Ostrava, 708 00 Ostrava, Czechia
[13] Department of Immunology, Public Health Institute in Ostrava, 702 00 Ostrava, Czechia
[14] Department of Epidemiology and Public Health, Faculty of Medicine, University of Ostrava, 70030 Ostrava, Czech Republic
[15] Department of Clinical Biochemistry, AGEL Hornická poliklinika s.r.o., 702 00 Ostrava, Czechia
[16] Department of Microbiology, Faculty of Medicine amd Dentistry, Palacky University Olomouc, 775 15 Olomouc, Czechia
[17] Department of Immunology, Faculty of Medicine and Dentistry, Palacky University Olomouc, 775 15 Olomouc, Czechia
[18] California Institute for Medical Research, 2260 Clove Dr., San Jose, CA 95128, USA
[19] Division of Infectious Diseases and Geographic Medicine, Stanford University School of Medicine, Stanford, CA 95128, USA
[20] Department of Analytical Chemistry, Faculty of Science, Palacký University, 771 46 Olomouc, Czechia

* Correspondence: radim.dobias@zuova.cz

Supplementary Data S1: Pentraxin 3 median concentration, cut-off, and Receiver Operating Characteristic analysis

S1.1. PTX3 median concentrations:

	Ptx3 (pg/mL)
**IPA**	4613
**CPA**	1746
**IPM**	4326
**FCol**	242
**BInf**	224

PTX3, pentraxin 3; IPA, Invasive Pulmonary Aspergillosis; CPA, Chronic Pulmonary Aspergillosis (including one patient with SAIA – Sub Acute Invasive Aspergillosis); IPM, Invasive Pulmonary Mucormycosis; Fcol, Fungal Colonization; BInf, pulmonary Bacterial Infections

S1.2. Receiver Operating Characteristic analysis:

Optimal cut-off for invasive fungal disease (in our case aspergillosis and mucormycosis) = **1653 pg/mL**, giving an area under the curve (AUC) of 0.9795

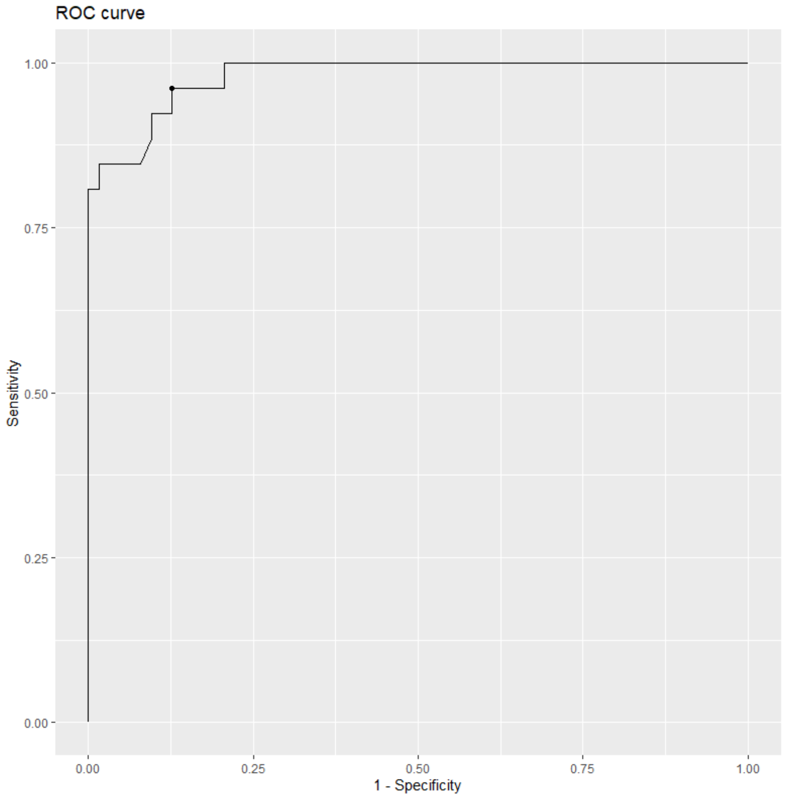

Optimal cut-off for invasive pulmonary aspergillosis = **2545 pg/mL,** giving an area under the curve (AUC) of 0.9991

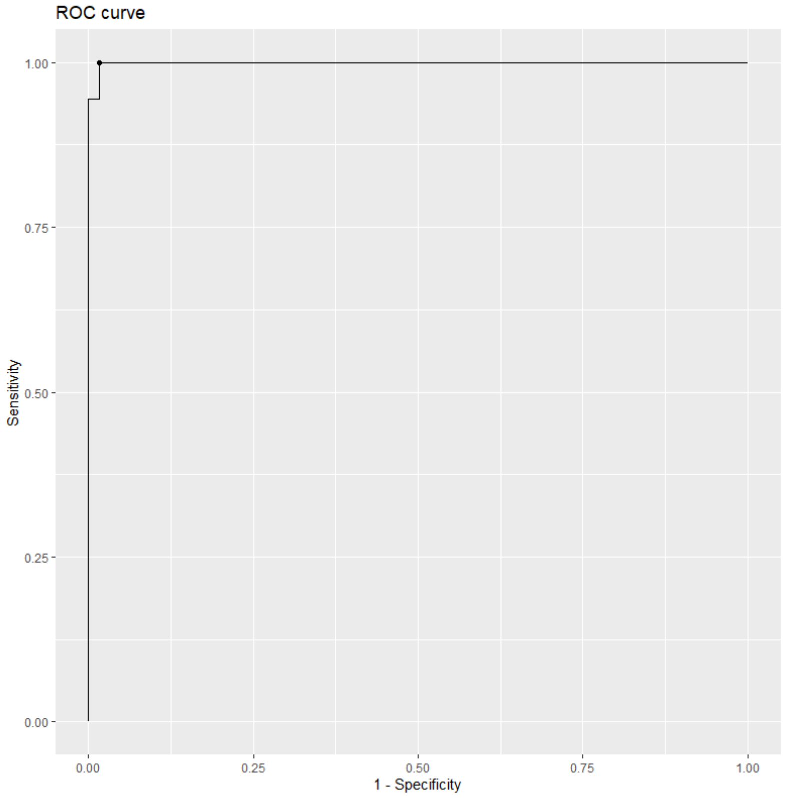

Supplementary Data S2: Pentraxin 3, triacetylfusarinin C (TafC) and ferricrocin (Fc) concentrations in bronchoalveolar lavage fluid from invasive pulmonary aspergillosis and invasive pulmonary mucormycosis patients

S2.1. Pentraxin 3, triacetylfusarinin C (TafC) and ferricrocin (Fc) concentrations in bronchoalveolar lavage fluid

IPA Patient no.	Ptx3 (pg/mL)	TafC/Crea (ratio)	Fc/Crea (ratio)
**1**	4733	ND	58.8
**2**	5000	72.7	ND
**3**	2545	370	18.3
**4**	5000	17.8	ND
**5**	4932	17.8	ND
**6**	4909	0.3	ND
**7**	4220	ND	ND
**8**	4639	ND	ND
**9**	4330	172.1	19.7
**10**	3902	ND	ND
**11**	4942	ND	ND
**12**	4547	ND	ND
**13**	4897	102.6	16.7
**14**	4880	34.4	ND
**15**	3496	0.3	ND
**16**	4588	209.6	36.2
**17**	4544	ND	ND
**18**	4116	194.1	ND
**IPM Patient no.**	**Ptx3 (pg/mL)**	**TafC/Crea (ratio)**	**Fc/Crea (ratio)**
**1**	1653	ND	ND
**2**	4352	ND	ND
**3**	4300	ND	ND
**4**	4920	ND	ND

S2.2. Diagnostic values of triacetylfusarinin C (TafC) and ferricrocin (Fc)

95% CI	TafC/IPM	TafC/IPA	Fc/IPA
**Sensitivity (%)**	2	61	0
**Specificity (%)**	100	98	100
**PPV (%)**	100	92	0
**NPV (%)**	7	90	22

IPA, Invasive Pulmonary Aspergillosis; IPM, Invasive Pulmonary Mucormycosis; Ptx3, Pentraxin 3; TafC, triacetylfusarinin C; Fc, ferricrocin; Crea, Creatinine; ND, not detected; PPV, Positive Predictive Value; NPV, Negative Predictive Value

Supplementary Data S3: Pentraxin 3 concentrations and galactomannan index values in bronchoalveolar lavage fluid samples from invasive pulmonary aspergillosis patients and analysis of the correlation between the two markers

Patient no.	Ptx3 (pg/mL)	GM (Positivity index)
**1**	4733	6.5
**2**	5000	7.20
**3**	2545	6.80
**4**	5000	7.60
**5**	4932	7.60
**6**	4909	2.44
**7**	4220	4.17
**8**	4639	8.30
**9**	4330	9.40
**9**	3902	1.26
**10**	4942	0.53
**11**	4547	0.85
**12**	3902	1.26
**13**	4942	0.53
**14**	4547	0.85
**15**	4897	8.41
**16**	4588	6.58
**17**	4544	0.61
**18**	4116	0.42

	Ptx3	GM
**Ptx3**	1.00000000	0.09411124
**GM**	0.09411124	1.00000000

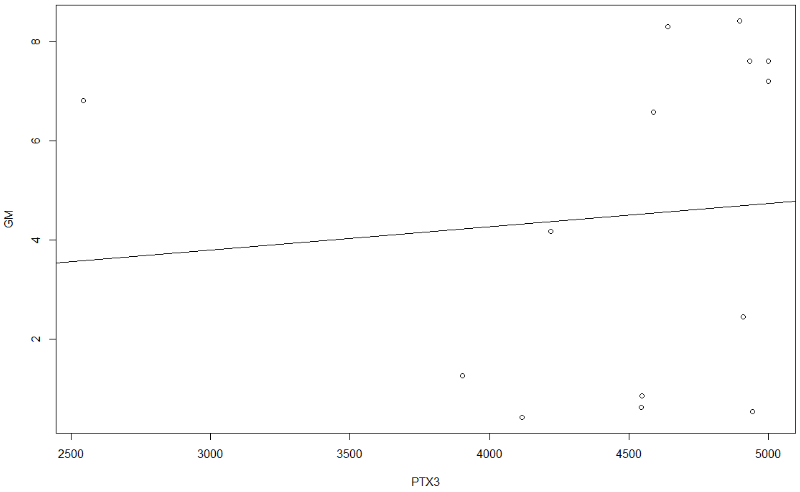

<u>**Pearson´s correlation between PTX3 and GM in BALF:**</u>

Linear association = 0.09411124

PTX3, pentraxin 3; GM, galactomannan

Supplementary Data S4: Correlation between galactomannan index values and concentrations of ferricrocin and triacetylfusarinine C in invasive pulmonary aspergillosis patients

	GM	Fc	TafC
**GM**	1.0000000	0.4428115	0.4459824
**Fc**	0.4428115	1.0000000	0.6736457
**TafC**	0.4459824	0.6736457	1.0000000

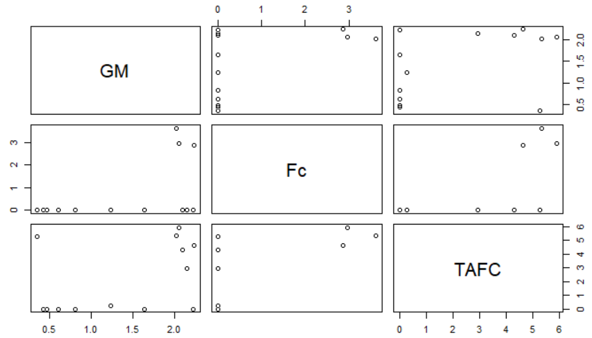

<u>Pearson´s correlation analysis for GM and Fc in BALF:</u>

Linear association = 0.4428115

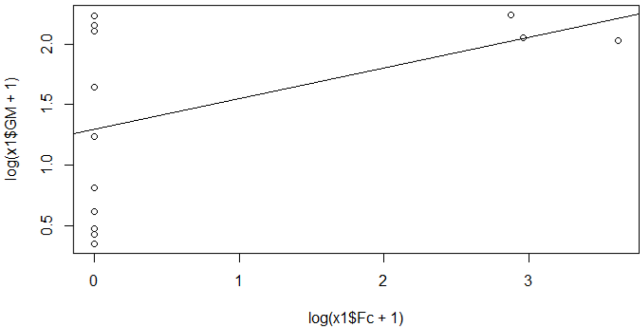

<u>Pearson´s correlation analysis for GM and TafC in BALF:</u>

Linear association = 0.4459824

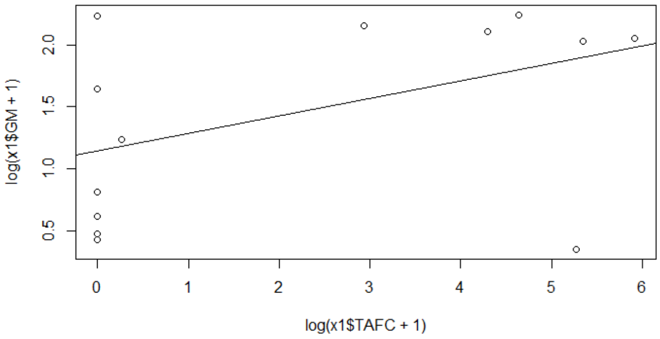

PTX3, pentraxin 3; GM, galactomannan; Fc, ferricrocin; TafC, triacetylfusarinine C
